# Clinical Lecture on Anæmia

**Published:** 1862-04

**Authors:** Thos. K. Chambers


### Selections.

### CLINICAL LECTURE ON ANÆMIA.

By THOS. K. CHAMBERS, M. D.

Reprinted from *Medical News and Library.*

Gentlemen: You will remember the corpse-like pallor, made more conspicuous by red hair, of a girl admitted this day fortnight into Victoria Ward. She smiled courteously, but was unable to rise from her bed. Her history is as follows:—

Margaret C., now aged 20, seems to have very good health in general, as is shown by her remembering that she had such an unimportant ailment as a pain in the right side when she was a school girl of seven years old. She seems to have been carefully brought up by a step father in a higher class of life; but three years ago she lost him, and had to go into service as a housemaid. For this work she was hardly strong enough, and, perhaps, too tenderly educated, and after eighteen months' trial, she gave it up, and was apprenticed to a Berlin-wool shop. In this place her mental superiority was apparently recognized, for she quickly became forewoman, with three girls under her, in a shop at Maidstone. She felt this responsibility a good deal, and also thought the closeness of the shop did not suit her, though it did not seem to make others ill. However, she retained a high, bright color in her face, for which she seems to have been somewhat admired, till nine months ago, when she began to lose it, and in a few weeks became as waxlike in hue as she is now. At first her appetite was large, and she always seemed in want of food; but after three months it failed, then ceased entirely, and she took a disgust to food. She had a good deal of pain in the epigastrium and to the left side of it, and also palpitation of the heart. Three months ago she spat up some blood, and had a little cough, which frightened her sadly. Three times during the nine months she has had attacks of low spirits, with crying, but does not appear at all hysterical now. The catamenia always were quite regular and sufficient till the commencement of the anæmia nine months ago, when they began to get scantier and scantier, and at last ceased entirely.

The urine is pale and watery, the stools scanty and steadily rare; but there is no sudden gush of bulky stools, diarrhœa alternating with constipation, or other indications of accumulation of feces in the intestines.

She expands her chest perfectly, and there are no abnormalities to give rise to a suspicion of pulmonary tubercle, at all events in such a quantity as to cause anæmia. There was a soft systolic murmur in the heart when she was agitated at first admission; but it went away after she had rested in bed five days.

Anæmia is found during life in a great number of the organic changes of tissues which you see in museums and lectures on morbid anatomy, and may discover by diagnosis. In other cases of equal importance and prominence it is absent. Very frequently, too, you find it in an extremely high degree in cases where you can discover no organic change in the solids at all, and where, from the transitory nature of the bloodlessness, there is reason to conclude that such organic changes really do not exist. Under this last category comes the patient who is the occasion of my present Lecture.

So anæmia or deficient redness in the blood, shows a deficiency of life in the ministers to that redness; either the supply of food is too small, or its assimilation is defective, in both cases either absolutely or relatively, to the existing demand.

In many instances it is easy enough to lay the finger upon the instrument of life which is to blame. We can detect without difficulty the causes at work—starvation, which anybody can understand leads to an absence of the organic matters made out of food; disease of stomach, in which the aliments are not prepared for assimilation; disease of liver and duodenum, producing the same result; disease of intestines, or their glands taking up no adipose matter especially, and so preventing cell growth; disease of the spleen or lungs, which physiological experiments, independent even of our observations of morbid phenomena, show to be answerable for the formation of new blood disks in a way yet unknown; mental derangement, care, disappointment, which so readily arrest the activity of the assimilating viscera; these agencies, and many more, are readily comprehended as causes of anæmia. But there are a considerable number of cases where nothing tangible of this sort is to be made out, yet where the paleness of the blood seen in the face, lips, tongue, or in a drop taken from a pricked finger, and evidenced by the faintness, weakness, palpitation, anasarca, amenorrhœa, etc., are even more more marked than where demonstrable lesion is to be found. So it is in the present instance. The young woman's history gives no reason to suspect any organic disease of the lungs or other organs, and the functions of life were fairly performed till she began to get pale and languid nine months ago. The want of red blood, which we look upon as the important feature in her case, attracted her attention also particularly, as she had previously had a fresh, high color. Then, after an interval amply sufficient to enable us to separate cause and effect, come the symptoms which I wish to notice as the consequence of anæmia. Causes, no doubt, they are in some instances, but here consequences. I mean the loss of appetite, impeded circulation, cessation of menses, hemorrhage from respiratory mucous membrane, and hysteria in a person unaccustomed to it.

The only explanation she can give of her loss of health is her having been employed in a shop less ventilated than she had been accustomed to, and having the responsibility of the concern thrown upon her. Alone neither would have been sufficient, as the shopwomen under her do not appear to have suffered from the air; while, on the other hand, women in retail business are not as a rule anæmic. But still I think that both together may perhaps be fairly saddled with the blame, for whilst the increased mental labor was increasing metamorphosis, the greater demand was not responded to by greater supply, but, on the contrary, assimilation was checked by the even moderate unwholesomeness of the respired air.

Of course, the not being able to trace deeper the anatomical cause arises from the imperfection of our knowledge, but it does not arise from neglecting to apply such knowledge as we possess to practical medicine. If we were to make an autopsy of this patient instead of curing her, we should in all probability find no more lesions in any of the tissues capable of accounting for the disease exhibited in the blood than we have already found. A fortnight ago Dr. Gull, Mr. Malton, and I examined the body of a gentleman who had died at forty-six of anæmia, and made separately microscopical investigations of portions of the several viscera. Nothing abnormal could we find in any part. The typical healthiness of all the tissues was very remarkable in a man of that age. There was not even a single adhesion of the pleura. I mention this in order that you may not lament the opacity of your patient's bodies, or suppose yourselves likely to learn how to treat them better if you could see their insides.

Anæmia, without obvious organic lesion, when properly treated, is a very curable condition, and this should still further reassure you, that you miss nothing by not being able to study its post-mortem pathology. For transitory and curable states leave but little foot-prints behind them for morbid anatomists. In a great majority of cases they depend upon the mucous membrane, of all the tissues in the body, the one most affected by mortuary changes.

To the mucous membranes I am disposed to attribute the condition in which we find our present patient. The two circumstances to which I have traced the illness both act directly or indirectly on this tissue. The mental exertion involved in an unusual responsibility thrown on a conscientious person would arrest the action of the involuntary muscles which carry along the mass of food through the alimentary canal. You know well the time your food is in leaving the stomach, if you are called to an important midwifery case just after a hearty meal; and several commercial and literary men have complained to me of attacks of vomiting (that is, temporary paralysis of the stomach), when they took dinner alone, and so were apt to let the mind dwell deeply on some interesting subject; and they have told me in wonder that they could dine out and eat and drink all sorts of rich things with impunity. They did not seem aware of the physiological value of frivolous conversation. At the same time that the moral causes thus impeded digestion, the unwholesomeness of the air in the close shop poisoned the mucous membranes, diminishing their vitality and causing them to be abnormally covered with a thick layer of mucus. Remember that, in spite of their name, it is not the business of mucous membranes to secrete mucus; the more perfect is their condition, the more favorable are the surrounding circumstances, the less they do so. From many persons' lungs not a drachm of expectoration is thrown up in a month, and the vast surfaces of the intestines and bladder are equally innocent of even microscopic traces of mucus in the typical health we desire to experience. It is only when the presence of some material agent diminishes their vitality that the mucous membranes exhibit on their surfaces that peculiar substance whence they take their appellation. And the greater the diminution of life, the greater the secretion; a slight cold in the head will be accompanied by slight catarrh, a severe one by excessive catarrh; and the nearer the approach to death, the greater it is, so that the death rattle, or overpowering collection of mucus in the bronchi, is a popular warning that all is over. Be careful not to look upon mucous secretion as augmented life; it is in fact a partial death.

Well, the poisoning air having covered these slowly moving mucous membranes with a thick tenacious coat, the entrance of alimentary substances into the veins and absorbents was impeded, and our patient starved in the midst of plenty. So all the usual signs of starvation followed. First, hunger—by no means a constant accompaniment of chronic deprivation of food, yet sometimes present as here; then anorexia, a much more frequent phenomenon; then paleness, languor, weariness, and pain in the stomach; then anasarca, and, in short, the other more marked symptoms of anæmia.

You must observe that the loss in those constituents of the body, which are of a nitrogenous chemical composition, is more marked than that in the hydrocarbonaceous fat. The reason is, partly, that the destruction of adipose vesicles is somewhat concealed by the saturation of the tissue with serum, which gives it a false plumpness—partly, that fat, being absorbable without much, if any alteration, is easier taken up than fibrin or albumen, which require a chemical solution before they can be absorbed. So that though starved, our patient looks but little emaciated.

All that I have said before, of course has for its end the treatment. My aim in anæmia is to introduce as quickly as I can the largest possible amount of—1, nitrogenous food; 2, iron; 3, chlorine. When I say "introduce" I do not mean "thrown in," or get swallowed, but assimilated in the system.

As regards the first, it is obvious that if I had written down ever so many "ordinary diets," a patient to whom the very sight of food was an abomination, would have gained nothing by it; she would simply have gone without. I directed therefore, no meals at all, and no solid food, but a cup of milk with some lime-water in it, to be given as medicine every two hours, and a pint of beef-tea in small, divided doses during the day. After two days she managed an egg also daily, and after twelve days of gradual additions of this sort, you will find her on full allowance of mutton chop, porter, beef-tea, and milk.

Iron is required to supply the new growth of red disks which we hope for, with their metallic constituent. You cannot get it into the system in any way so quickly as the mistura ferri composita of the London Pharmacopœia. Large doses of the more soluble salts have an action on the mucous membranes which not only prevents them being taken up, but also arrests the digestion of food. Evidence of the latter is found in loss of appetite and feverishness, and of their own rejection in the blackening of the stools much sooner than by the form I have approved of. So in spite of the elegant preparations which are constantly put before us, as recommended by the solubility, such as the chloride, acetate, citrate, phosphate and other salts of iron, I prefer the unchemical mixture. It seems as if the carbonate which is preserved from decomposition by the sugar, and the finely-divided oxides diffused through the thick liquid were peculiarly easy of solution in the water saturated with salts and carbonic acid, which (and not pure water) we must remember is the solvent to be considered.

I have found that some cases which did not improve so quickly as I could wish under the above treatment, made a sudden start of improvement when to it, was added the administration of chlorine in the form of warm hydrochloric acid baths. More iron is taken up—the blackening of the feces ceases, and therefore perhaps it may be that the presence of more acid in the system attracts more of the metal. But in a few cases I tried for experiment the hydrochloric acid baths alone, and even then it was beneficial, seeming to confer muscular strength like what are commonly called tonic drugs. I cannot but think, therefore, that it supplies a distinct want in the system, that it is a directly restorative medicine in anæmia.

Nor is it difficult to make this empirical observation accord with rational pathology. In anæmia the blood is more watery than natural; the proportion is deficient, not only of organic matters, but of salts. Chloride of sodium is the most important of these, and the supply of one of the constituents of this material we may reasonably imagine is an aid to the renewal of life, which is the end of all medication.

Besides the above named medicines, you will see, I have ordered Pil. aloes cum myrrh, gr. iv. omni nocte sumenda. Now, do not suppose that this is ordered merely as a purgative, and that any other purgative would do as well. On the contrary, most purgatives do harm in anæmia. Gamboge, castor-oil, sulphate of magnesia, colocynth, mercury, and several others which produce serous elimination and augment secretion generally, would do harm just in proportion to their activity. It seems established by the experiment of making them act as purgatives when injected into the circulation, that their soluble principles have a destructive agency over the blood; whereas the soluble alkaloid in aloes (aloine) is, in fact, a bitter tonic, and the purgative power of the drug resides in its insoluble resin. * Its action is very slightly

* "Headland on the Action of Medicines," p. 331; and Robiquet in *Journal de Pharmacie*, April, 1856.

eliminative—in moderate doses it only slightly augments the solid brown excreta of the colonic glands, and produces feces feculent in smell and of consistent form; whilst at the same time it restrains, by its bracing bitter, the formation of mucus, as you may clearly see by its action on moist piles, how it dries them up and makes them smart. And by the more vigorous peristaltic action and by the solid mass passed along the gut, the already existing mucus is cleared away. Aloes, therefore, is employed strictly as a clearer of the intestinal, especially of the colonic, membrane. It is joined with myrrh, partly to divide it minutely, and make a small dose go further, and partly to get the advantage of the extra resin.

November 28. A fortnight ago I lectured about an anæmic patient. She was then showing tendency to lose her title to the name, and now she certainly cannot claim it, and has earned our confidence in the statement that her natural hue is rosy. She leaves the Hospital to-day, having manufactured enough red disks to color her blood throughout very sufficiently.

What amount of manufacturing industry does this show? Let us reckon. She weighs 8 stone, or 1792 ounces; of this $_{7}$ths, or 512 ounces is blood; and of this blood $_{1000}$, that is to say, 60 ounces should be red globules. Now the analyses of M. M. Andral and Gavarret show that in cases of anæmia of at all a marked character (as this was), we may expect, at least, three-quarters of the red disks to disappear, so that when she came into the Hospital it may be fairly assumed that she did not possess above 15 ounces; and now I think with equal fairness she may be assumed to have got up to 45, which is conceding that she still wants a quarter of perfect health. By this rekoning she must have made 20 ounces of red blood disks; that is, the most important organic constituent of upwards of150 ounces of blood, in a month!

Mark the power of renewal which the human body has under favorable circumstances, and learn from this not only the curability of anæmia when it is a disease, but also the facility of repairing artificial loss of blood when it is employed as a remedy

Only note this, that *if the loss is to be repaired, the means of repair must be given.*

---

Dr. Pamard, mayor of Avignon, has just been elected to a seat in the House of Representatives of Paris.—*London Lancet.*

